# Supplementary material for: Pre-COVID-19 knowledge, attitude and practice among nurses towards infection prevention and control in Bangladesh: A hospital-based cross-sectional survey
Source: PLoS One. 2022 Dec 1;17(12):e0278413. doi: 10.1371/journal.pone.0278413 (PMC9714721; doi:10.1371/journal.pone.0278413)
Supplement: Attachment — Submitted filename: Response to Reviewers.docx [file pone.0278413.s002.docx]

Date: October 30, 2022

To

The Editor in Chief

PLOS ONE

Subject:	Incorporation of reviewers’ comments and resubmission of the manuscript (ID PONE-D-22-12028) titled "Pre-COVID-19 knowledge, attitude and practice among nurses towards infection prevention and control in Bangladesh: A hospital-based cross-sectional survey"

Dear Editor:

Thank you very much for your email regarding the incorporation of reviewers' comments on our manuscript (PONE-D-22-12028) titled "Pre-COVID-19 knowledge, attitude and practice among nurses towards infection prevention and control in Bangladesh: A hospital-based cross-sectional survey."

I am pleased to resubmit the revised version. We (Author & Co. Authors) tried our best to concentrate on comments/suggestions made by both reviewers. We appreciated both of the reviewers for their excellent comments. All comments/suggestions have been incorporated into the manuscript as well.

We hope that the incorporated comments/suggestion will satisfy the reviewers and editor and expect the continuation of the production of our manuscript.

**Editor comments and authors' response:**

**Editor comments**:**The first issue** relates to the knowledge item about glutaraldehyde sterilization. In the original manuscript, responses to the survey item “A contaminated item soaked in glutaraldehyde for 10 minutes is sterilized” were listed as 29% “Correct” and 71% “Incorrect”. Reviewer #2 had commented that “In line 297 you state that "it is recommended that the use of glutaraldehyde for at least 10 minutes for sterilizing surgical and ward instruments". This is not correct. For chemical sterilization it is required 8 to 10 hours of exposition of glutaraldehyde.”

The text in the original manuscript’s results section (lines 202 – 206) stated, “On the contrary, a low percentage of correct answers were found for knowledge questions related to the resources needed to comply with infection prevention guidelines (23.3%), bending used needles (4.3%), **drenching in glutaraldehyde for 10 minutes for decontamination (29.0%)** and compliance with IPC guideline even in heavy workload (40.3%)”.

This indicates that the responses to this survey item were originally considered to be correct if a respondent agreed that an item could be sterilized if soaked in glutaraldehyde for 10 minutes. In the revised manuscript, the authors added the following to the discussion (lines 298-300), “It is recommended that the use of glutaraldehyde for 10 hours at 20-35°C for sterilizing surgical and ward instruments is a recommended approach (34). In our study, only 29% of the nurses knew how to properly use this chemical.” However, the survey item still contains the original statement “A contaminated item soaked in glutaraldehyde for 10 minutes is sterilized” and the response rates are the same as they were in the previous version. There is a major concern about what time frame the survey item originally stated (10 minutes versus 10 hours) and why the percentage of “correct” and “incorrect” responses remains the same in the revised manuscript. If a respondent answered that 10 minutes was not adequate then they actually had the right answer, which would indicate that 71% of respondents were “Correct” instead of “Incorrect”. The authors must address this issue and assure that those results are presented consistently and accurately throughout the manuscript.

**Authors’ response:**

Thank you so much for your kind observation. Actually, we used the question “A contaminated item soaked in glutaraldehyde for 10 minutes is sterilized,” and those who answered ‘no’ was correct, and 29% answered correctly. However, we have presented this finding in the manuscript correctly. “A contaminated item soaked in glutaraldehyde for 10 hours at 20-35°C is sterilized”. We have also updated the result, discussion, and table sections accordingly.

**Editor comments: The second issue** that needs to be addressed is the survey item “All staff working in the hospital should follow the IPC instructions and carefully deal with the patient is considered a potential infection control practice”. The translation of this item to English is confusing and thus the meaning of the item is unclear.  The first part of the item (All staff working in the hospital should follow the IPC instructions) does not need to be changed but the second part of the item (and carefully deal with the patient is considered a potential infection control practice) needs to be edited for clarity.

**Authors’ Response:**

Thank you for the observation. We have edited the question and made it clear for understanding in the revised version of the manuscript.
